# Loss of Catalytically Inactive Lipid Phosphatase Myotubularin-related Protein 12 Impairs Myotubularin Stability and Promotes Centronuclear Myopathy in Zebrafish

**DOI:** 10.1371/journal.pgen.1003583

**Published:** 2013-06-20

**Authors:** Vandana A. Gupta, Karim Hnia, Laura L. Smith, Stacey R. Gundry, Jessica E. McIntire, Junko Shimazu, Jessica R. Bass, Ethan A. Talbot, Leonela Amoasii, Nathaniel E. Goldman, Jocelyn Laporte, Alan H. Beggs

**Affiliations:** 1 Genomics Program and Division of Genetics, The Manton Center for Orphan Disease Research, Boston Children's Hospital, Harvard Medical School, Boston, Massachusetts, United States of America; 2 Department of Translational Medicine and Neurogenetics, Institut de Génétique et de Biologie Moléculaire et Cellulaire, Inserm U964, CNRS UMR7104, Université de Strasbourg, Collège de France, Chaire de Génétique Humaine, Illkirch, France; The Jackson Laboratory, United States of America

## Abstract

X-linked myotubular myopathy (XLMTM) is a congenital disorder caused by mutations of the myotubularin gene, *MTM1*. Myotubularin belongs to a large family of conserved lipid phosphatases that include both catalytically active and inactive myotubularin-related proteins (i.e., “MTMRs”). Biochemically, catalytically inactive MTMRs have been shown to form heteroligomers with active members within the myotubularin family through protein-protein interactions. However, the pathophysiological significance of catalytically inactive MTMRs remains unknown in muscle. By *in vitro* as well as *in vivo* studies, we have identified that catalytically inactive myotubularin-related protein 12 (MTMR12) binds to myotubularin in skeletal muscle. Knockdown of the *mtmr12* gene in zebrafish resulted in skeletal muscle defects and impaired motor function. Analysis of *mtmr12* morphant fish showed pathological changes with central nucleation, disorganized Triads, myofiber hypotrophy and whorled membrane structures similar to those seen in X-linked myotubular myopathy. Biochemical studies showed that deficiency of MTMR12 results in reduced levels of myotubularin protein in zebrafish and mammalian C2C12 cells. Loss of myotubularin also resulted in reduction of MTMR12 protein in C2C12 cells, mice and humans. Moreover, XLMTM mutations within the myotubularin interaction domain disrupted binding to MTMR12 in cell culture. Analysis of human XLMTM patient myotubes showed that mutations that disrupt the interaction between myotubularin and MTMR12 proteins result in reduction of both myotubularin and MTMR12. These studies strongly support the concept that interactions between myotubularin and MTMR12 are required for the stability of their functional protein complex in normal skeletal muscles. This work highlights an important physiological function of catalytically inactive phosphatases in the pathophysiology of myotubular myopathy and suggests a novel therapeutic approach through identification of drugs that could stabilize the myotubularin-MTMR12 complex and hence ameliorate this disorder.

## Author Summary

Congenital myopathies are a group of heredity diseases characterized by muscle weakness and impaired locomotion that manifest in both children and adults. X-linked myotubular myopathy (XLMTM) is a subtype of congenital myopathy that predominantly affects males and is caused by mutations in the myotubularin (*MTM1*) gene. To date, more than 200 pathogenic mutations have been identified in *MTM1*. However, no effective therapy is available to treat patients presenting with XLMTM. This is largely due to a lack of understanding of molecular processes perturbed in the XLMTM disease state, thereby limiting the availability of suitable therapeutic targets. In this study, we show that catalytically inactive MTMR12 interacts with myotubularin in skeletal muscle. This complex formation is required to provide stability to myotubularin in the normal functioning of skeletal muscle and these interactions appear to be disrupted in XLMTM. This work therefore offers a novel direction for therapy development, both in XLMTM and other genetic diseases, by identifying crucial protein interactors of disease-causing proteins whose complexes might be stabilized in the disease state to restore normal function.

## Introduction

X-linked myotubular myopathy (XLMTM) is a congenital disorder caused by mutations of the *MTM1* gene that encodes myotubularin [1], [2]. Affected males are born with severe generalized hypotonia and weakness of skeletal muscles with respiratory insufficiency. In majority of cases the disease is fatal within the first months of life, but a proportion of affected males survive into their teens or beyond yet are non ambulant and require ventilatory support. Histopathologically, affected muscle fibers exhibit hypotrophy with a large number of centrally placed nuclei in a high proportion of myofibers. Thus, XLMTM is considered a subtype of centronuclear myopathy (CNM) [3]. *MTM1* encodes a 3′-phosphoinositide (PtdIns3*P*) lipid phosphatase that catalyzes the dephosphorylation of phosphatidylinositol-3-phosphate (PtdIns3*P*) and phosphatidylinositol-3,5-bisphosphate PtdIns(3,5*P)_2_* [4], [5], [6], [7]. Phosphoinositides (PIs) are critical for a variety of physiological processes, including cell proliferation, cell death, motility, cytoskeletal regulation, intracellular vesicle trafficking, autophagy and cell metabolism [8]. PtdIns3*P* and PtdIns(3,5)*P*_2_ are both important regulators of membrane trafficking [9]. Similar to humans, loss of myotubularin function in animal models also results in clinico-pathological symptoms similar to myotubular myopathy signifying evolutionary conservation of mechanisms involving myotubularin [10], [11], [12], [13].

Myotubularin is the prototypic member of one of the largest and most conserved protein lipid phosphatase subfamilies in eukaryotes, the myotubularin-related proteins (MTMRs) [14], . There are nine catalytically active members of the MTMR family, and six members whose catalytic sites have been inactivated through missense changes (designated myotubularin and MTMR1-14). While several active members are known to play crucial roles in physiology and human diseases presumably related to loss of their enzymatic activities, the cellular functions of catalytically inactive members are still poorly understood. Catalytically inactive MTMR family members are highly conserved and retained in vertebrate genome from teleosts to humans during evolution [16] suggesting that these inactive members have important roles in development and diseases independent of their enzymatic activities, as functionally redundant family members are often lost during evolution [17]. This is further evident by a recent study that showed that catalytically inactive form of myotubularin could ameliorate many of the structural abnormalities seen in *Mtm1* knockout mice [18].

Myotubularins form homo- and heteroligomers with themselves and other members of the MTMR family, and the catalytically inactive MTMRs are thought to largely form oligomers with active family members [14], [19]. These interactions appear to regulate the sub-cellular localization or catalytic activity of the active members of the myotubularin family. *In vitro*, catalytically inactive MTMR5 and MTMR9 modulate the enzymatic activity of their interacting catalytically active partners, MTMR2, and MTMR6 and MTMR8, respectively [20], [21], [22]. Functional evidence for *in vivo* significance of these interactions comes from genetic studies in humans that show that mutations of either the catalytically active MTMR2 or its catalytically inactive binding partner MTMR13 result in similar forms of Charcot-Marie-Tooth disease [23], [24], [25], [26]. Similarly, mutations in either of the binding partners MTMR2 (catalytically active) or MTMR5 (catalytically inactive) lead to defective spermatogenesis in mice, suggesting the biological importance of these interactions [27], [28].

Myotubularin-related protein 12 (MTMR12), previously sometimes referred to as 3-phosphatase adaptor protein (3-PAP), is a catalytically inactive member of myotubularin family that interacts with MTM1 and MTMR2 to regulate their sub-cellular localization in *in vitro* studies of non-muscle cells [29], [30]. The *in vivo* significance of such interactions remains unknown, especially with reference to disease process in myotubular myopathy that primarily affects the skeletal muscles due to lack of myotubularin function [10]. Protein-protein interactions are critical components of almost every biological process and any disruption of these networks leads to pathological conditions resulting in related diseases [31], [32]. Therefore, a proper understanding of myotubular myopathy necessitates a comprehensive knowledge of the molecular mechanisms that govern such processes. We have employed zebrafish (*Danio rerio*) as a vertebrate animal model to understand the role of MTMR12 as a molecular regulator of myotubularin. Zebrafish are an excellent vertebrate model to study skeletal muscle development and disease due to high genomic synteny and similar clinico-pathological changes as seen in the human myopathies. Our studies revealed that deficiency of MTMR12 in zebrafish results in myopathy and impaired motor function similar to that caused by loss of myotubularin. Our data suggest MTMR12 affects muscle function primarily by regulating the stability of myotubularin *in vivo*. Studies in cells, mice and humans further implicate MTMR12 as an important player in the pathophysiology of XLMTM.

## Results

### MTMR12 interacts with myotubularin *in vitro* and *in vivo* in skeletal muscles

**Figure 1:**
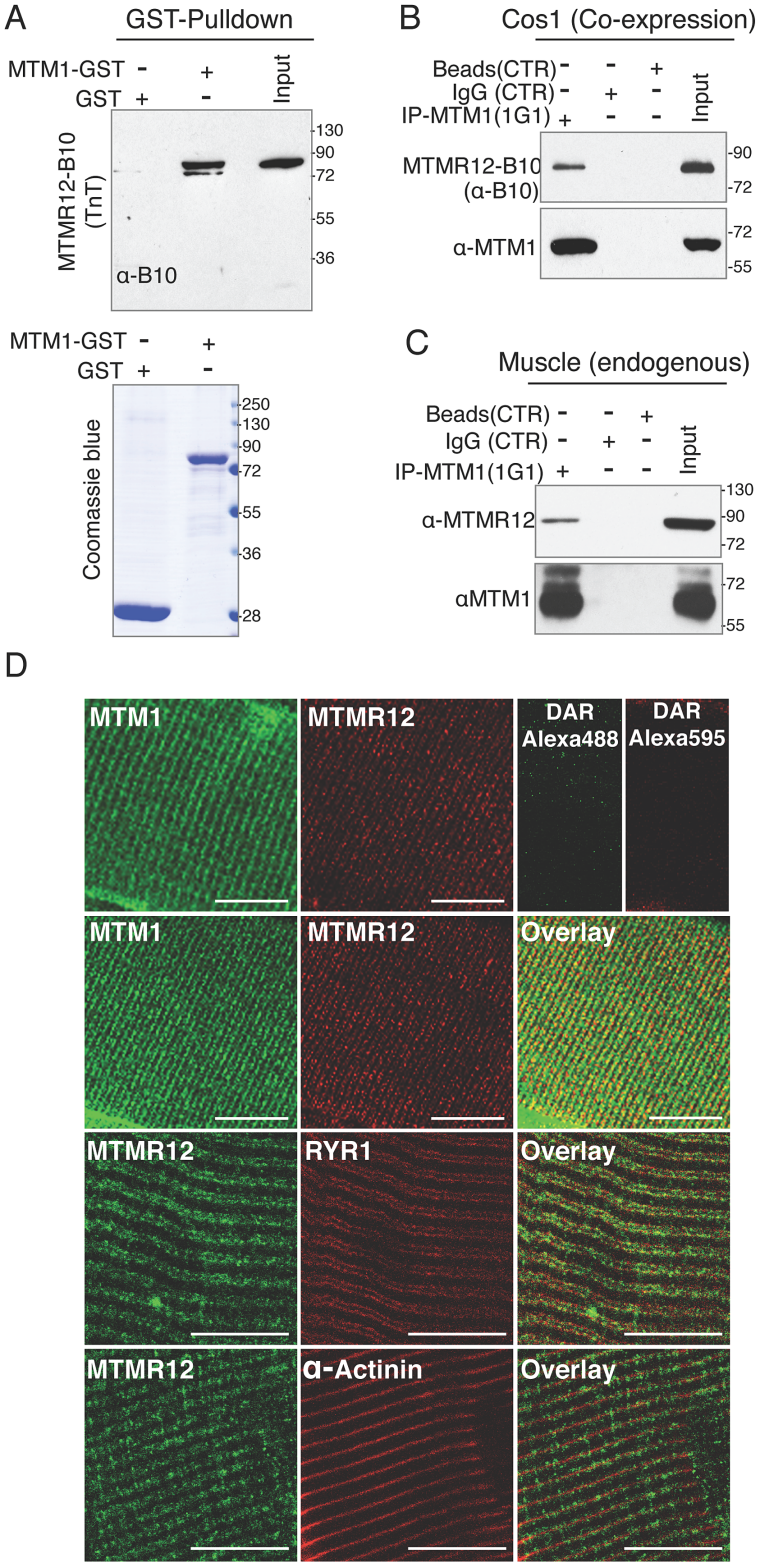
Protein-protein interactions between myotubularin and MTMR12 proteins. (A) GST pull down of MTM1-GST recombinant protein with MTMR12 showed a direct interaction between the two proteins (above). Coomassie blue stained gel (below) showing GST and MTM1-GST protein used in the pull down (B) Co-IP experiments from Cos-1 transfected cells with MTM1 and MTMR12-B10 constructs showing the interaction of the two proteins in the cellular context *in-vivo* (C) MTMR12 co-immunoprecipitates with myotubularin (using 1G1 monoclonal antibody) in mouse muscle lysates revealed with anti-MTMR12 polyclonal antibody (upper panel) and with the 2827 polyclonal anti-myotubularin (bottom panel). (D) Confocal microscopic immunofluorescence studies of longitudinal frozen sections of skeletal muscle from mouse tibialis anterior muscle with sarcomeric markers. Individual immunostaining with 2827 anti-myotubularin or anti-MTMR12 showed similar striated localization in skeletal muscle (top panel). Double-immunostaining with both proteins showed a co-localization of myotubularin and MTMR12 to Triads and partial co-localization with the ryanodine receptor, RyR1, a sarcoplasmic reticulum marker, but not with α-actinin, a Z-line marker. Scale bar = 10 µm.

MTMR12 has been shown to interact with myotubularin and form oligomers in K562 and Cos7 cells [30]. To investigate if the binding between these proteins is a direct consequence of protein-protein interactions or is an indirect interaction mediated through another binding partner, GST-pull down assay was performed *in vitro* (Figure 1A). Human myotubularin full-length protein was expressed as a GST fusion protein in *E. coli* (MTM1-GST). Equivalent amounts MTM1-GST or control GST protein bound to glutathione beads were incubated with *in vitro* synthesized MTMR12 protein with a B10 tag (MTMR12-B10). MTM1-GST protein pulled down MTMR12-B10 protein whereas no interaction was observed with the GST alone, suggesting myotubularin and MTMR12 interact with each other by direct protein-protein interactions (Figure 1A). The interaction between myotubularin and MTMR12 was also examined in the Cos1 cell line. MTM1 and MTMR12-B10 were over-expressed in Cos1 cells and cell extracts were immunoprecipitated by an antibody against myotubularin (monoclonal, 1G1). This resulted in co-immunoprecipitation of MTMR12-B10 with myotubularin but not with control IgG or empty beads confirming that MTMR12 also interacts with myotubularin in the cellular context as reported earlier (Figure 1B) [30].

Mutations in *MTM1* result in XLMTM, which is a congenital myopathy that primarily affects the skeletal muscles in human patients and animal models. Therefore, the interaction between myotubularin and MTMR12 was investigated in skeletal muscles. Co-immunoprecipitation was performed using protein extracts from murine skeletal muscle (tibialis anterior) using an anti-myotubularin monoclonal antibody (1G1) (Figure 1C). Antibody against myotubularin co-immunoprecipitated endogenous MTMR12 protein showing that myotubularin and MTMR12 interact in skeletal muscles. Control IgG or empty beads failed to immunoprecipitate MTMR12 suggesting the specificity of interactions between myotubularin and MTMR12. *In vivo* interaction between these proteins was also investigated by immuno-colocalization of myotubularin and MTMR12 proteins in murine skeletal muscles. Immunofluorescence showed a striated expression of endogenous MTMR12 in mouse tibialis anterior skeletal muscle similar to the pattern of myotubularin staining, and double immunolabeling revealed that these colocalize at the resolution of confocal microscopy (Figure 1D). In addition, partial co-localization of MTMR12 was observed with ryanodine receptors, a sarcoplasmic reticulum marker. The expression of MTMR12 was restricted to triads as no co-localization was observed with α-actinin at Z-lines (Figure 1D). Therefore, a combination of GST pull down, co-immunoprecipitation and co-localization studies support the hypothesis that myotubularin and MTMR12 interact with each other in skeletal muscle.

### *Mtmr12* knockdown results in skeletal muscle myopathy in zebrafish

**Figure 2:**
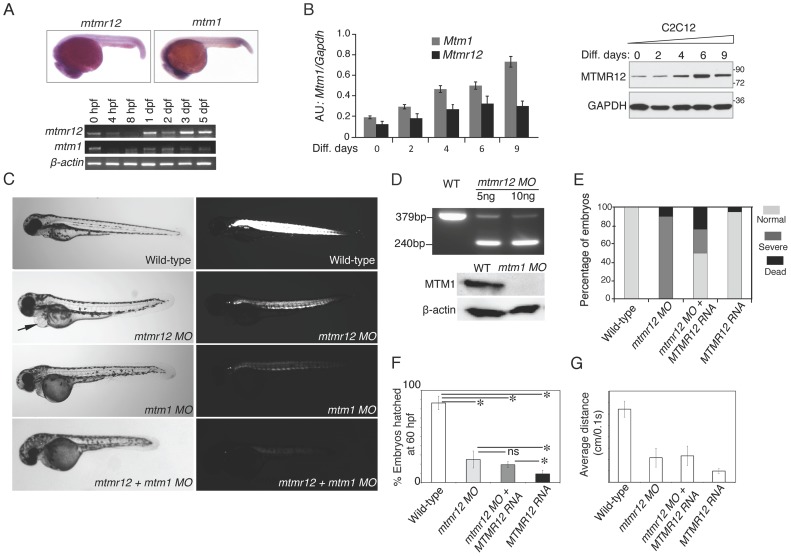
Expression patterns and morpholino-based knockdown of *mtmr12* in developing zebrafish. (A) Whole mount in-situ hybridization detected ubiquitous expression of *mtmr12* and *mtm1* transcripts in zebrafish embryos at 1 dpf (above). Below is RT-PCR analysis of *mtm1* and *mtmr12* expression during zebrafish development using RNA extracts from whole zebrafish embryos at indicated developmental timepoints. (B) Synergistic expression level of *Mtm1* and *Mtmr12* transcripts and protein at indicated time points of C2C12 differentiation (0–9 days) monitored by RT-quantitative PCR (corresponding histogram, *P≤0.05) and by western blot analysis (right panel). (C) Live embryos at 3 dpf injected with control, *mtmr12*, *mtm1* or both *mtmr12* and *mtm1* morpholinos in normal (left) and polarized lights (right). *mtmr12* morphant fish showed a dorsal curvature in skeletal muscle and reduced birefringence in polarized light similar to *mtm1* morphant embryos. *mtmr12* morphant fish also exhibited pericardial edema (arrow). *mtmr12-mtm1* double knockdown fish exhibited smaller size and reduced birefringence relative to *mtm1* or *mtmr12* alone morphant fish. (D) *mtmr12* mRNA levels in *mtmr12* morphant zebrafish following injection of two different amounts of morpholino (indicated below, upper panel). In *mtm1* morphant fish, no residual myotubularin was observed showing that *mtm1* morpholinos are completely penetrant to the limits of detection for western blotting. (E) Over-expression of human *MTMR12* mRNA rescued small body length and skeletal muscle abnormalities observed in *mtmr12* morphant embryos. (F) Quantification of the chorion hatching at 60 hpf. The number of embryos was quantified in three independent clutches (number of embryos in each clutch = 90–120). (G) Quantification of touch evoke response at 3 dpf (n = 5–8 embryos were assayed in each morpholino group).*P≤0.01.

To understand the function of MTMR12 *in vivo*, zebrafish was used as a vertebrate animal model. The zebrafish *mtmr12* gene encodes a protein of 736 amino acids that is 69% similar and 52% identical with the human MTMR12 protein. The expression of *mtmr12* was analyzed by whole mount in-situ hybridization and RT-PCR in developing zebrafish embryos. Whole mount in-situ hybridization showed that *mtmr12* transcripts were expressed ubiquitously in developing eyes, brain, heart and skeletal muscles at 1 day post fertilization (dpf) (Figure 2A). This ubiquitous expression of *mtmr12* was similar to the ubiquitous expression of *mtm1* transcripts at 1 dpf. Similar to the zebrafish gene, human *MTMR12* transcripts have also been found to express in all organs [33]. RT-PCR analysis further showed similar temporal expression of *mtm1* and *mtmr12* during zebrafish development (Figure 2A). RT-PCR revealed that *mtmr12* and *mtm1* were first detected as maternal transcripts at the 1 cell stage. Expression of these maternal transcripts decreased during gastrulation and zygotic expression appeared around 8 hours post fertilization (hpf). This zygotic expression persisted in all developmental stages tested (until 5 dpf). In murine muscle cells, *Mtmr12* transcripts were detected during proliferation as well as differentiation stages in C2C12 cells (Figure 2B). The expression of *Mtmr12* transcripts increased steadily during differentiation followed by a decrease in late differentiation. Comparison to *Mtm1* expression revealed a similar expression pattern with a continuous increase with the progression of differentiation. Similar to the mRNA expression, MTMR12 protein expression was also highest during differentiation (Figure 2B). Similar expression of *mtmr12* and *mtm1* during zebrafish development and in muscle cell differentiation suggests that they may be involved in similar physiological processes.

To investigate *in vivo* functions of the *mtmr12* gene in zebrafish, anti-sense morpholino technology was employed to achieve functional gene knockdown (Figure 2C–E). Morpholinos were designed to disrupt either the translation or splicing of *mtmr12* transcripts. Knockdown using either a splice site-morpholino targeting the exon3-intron3 junction or the translational (ATG) morpholino resulted in similar phenotypes at relatively low morpholino concentrations (3.5–5.0 ng), suggesting the specificity of their action. Microinjection of 1cell embryos with splice site morpholinos resulted in mis-splicing and exclusion of exon 3 from the mature mRNA as detected by RT-PCR assay (Figure 2D). *Mtmr12* knockdown fish (*mtmr12* morphants) are smaller in size compared to controls and exhibited a dorsal curvature through the back and tail, instead of the normal flat dorsum, similar to *mtm1* knockdown fish (Figure 2C). Axial skeletal muscles of zebrafish embryos were examined using a birefringence assay that involves examination of axial skeletal muscles of live zebrafish embryos using polarized filter microscopy. Skeletal muscles of *mtmr12* morphant embryos showed a reduced birefringence in comparison to the control morpholino injected fish suggesting a defect in skeletal muscle organization. Several *mtmr12* morphants also displayed pericardial edema. Similar phenotypes were obtained with both translational as well as spice-site morpholinos suggesting the specificity of morpholino knockdown. None of the commercially available antibodies showed reactivity to zebrafish MTMR12 protein, therefore, the rest of the studies were performed with the splice-site blocking morpholino against exon3-intron3 junction as mRNA knockdown could be assayed. To further validate the specificity of these phenotypes and to rule out any off-site targeting effect of morpholinos, mRNA rescues were performed. Human *MTMR12* mRNA was co-injected with *mtmr12* morpholino in zebrafish embryos. Over-expression of *MTMR12* mRNA in *mtmr12* morphant fish resulted in a rescue of phenotypes seen in *mtmr12* morphant fish indicating the specificity of morpholino targeting (Figure 2E).

To understand if the interacting partners myotubularin and MTMR12 are involved in similar biological processes or have functions that are independent of each other, double knockdowns were performed. Double knockdown fish were smaller in size then either *mtm1* or *mtmr12* morphant fish. Polarized live microscopy of morphant fish also showed that birefringence of *mtmr12-mtm1* double morphant zebrafish muscle was lower than in either *mtm1* or *mtmr12* morphant fish suggesting a severe muscle phenotype. The exacerbated phenotypes of double knockdown fish suggests that in addition to regulating similar biological processes these genes may be involved in different processes independent of each other.

### MTMR12 deficiency results in impaired motor function in zebrafish

To understand the consequences of MTMR12 deficiency on motor function, behavioral analysis of zebrafish embryos was performed. During early development, zebrafish embryos hatch out of their chorions by regular contractions of their skeletal muscles. Typically, approximately 86±7.4% of control embryos hatch by 60 hpf. In contrast, only 25±9.3% of *mtmr12* morpholino-injected embryos hatched by this time, consistent with a continued decrease in motor activity early in development (P<0.01, n = 100–170) (Figure 2F). *Mtm1* morphant embryos displayed similar behavior as *mtmr12* morphants (19.3±3.39, n = 100–150, P<0.001). Knockdown of both MTMR12 and myotubularin resulted in a significant decrease in hatching behavior in comparison to the *mtmr12* alone morphant fish. (9.33%±4.02, P<0.001, n = 100–130).

*Mtmr12* morphant fish were largely immotile and their touch evoked response was blunted; instead of rapidly swimming out of the field of view like control fish (6.44±0.712 cm/0.1 sec), they twitched and only moved several lengths when stimulated with a needle (2.16±(2.832 cm/0.1 sec), suggesting a significant degree of overall muscle weakness (Movie S1, Figure 2G). This decrease in touch-evoked response was similar to that of *mtm1* morphant fish (2.32±0.855 cm/0.1 sec) (Movie S1, Figure 2G). The touch-evoked response was also evaluated in *mtm1-mtmr12* double morphant fish. In comparison to either *mtm1* or *mtmr12* knockdown embryos, double morphant embryos showed a greater degree of reduction in touch-evoked escape response (0.98±0.230 cm/0.1 sec) (Movie S1, Figure 2G). The delayed chorion hatching and diminished touch-evoked escape behaviors showed that *mtmr12* is required for normal motor function in zebrafish.

### MTMR12 deficiency results in sarcomere disorganization with central nucleation in skeletal muscles

**Figure 3:**
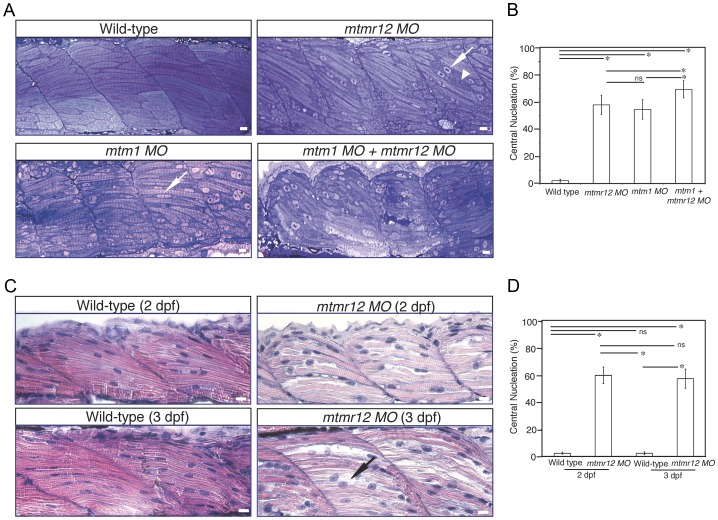
Abnormal histology of MTMR12-deficient zebrafish. (A)Toluidine blue stained longitudinal sections of skeletal muscle in control and morphant fish at 3 dpf. In comparison to the control fish, *mtmr12* morphants showed disorganized myofibers (arrowhead) with central nucleation (arrow), similar to histological changes observed in the skeletal muscle of *mtm1* morphant fish (arrow). Knockdown of both *mtm1* as well as *mtmr12* results in severe muscle disorganization greater than seen in *mtm1* or *mtmr12* alone morphants. (B) Centrally nucleated myofibers were quantified. Serial sections from 3–4 different embryos were analyzed and the relative number of centrally nucleated fibers in the middle somites (10–13) were counted. (C) Hematoxylin and Eosin staining of *mtmr12* morphant zebrafish at different time points. An increase in sarcomeric disorganization was observed at 3 dpf in comparison to 2 dpf in *mtmr12* morphants (arrow) (D) Centrally nucleated myofibers were quantified. Serial sections from 6 different embryos were analyzed and the relative number of centrally nucleated fibers in the middle somites (10–13) were counted. Scale bar = 10 µm.

To study the effect of MTMR12 deficiency on skeletal muscle structure, ultrathin toluidine blue-stained longitudinal sections of control and *mtmr12* knockdown fish were examined at 3 dpf. Histological examination of control muscle showed well-organized myofibers with elongated nuclei that were localized to the periphery of muscle fibers (Figure 3A). In *mtmr12* knockdown fish, areas lacking sarcomeric organization were observed (Figure 3A, arrowhead). Moreover, occasional rounded, central nuclei were seen in skeletal muscles of the morphant fish but were absent in the control fish (Figure 3A, arrow). Remarkably, the sarcomeric disorganization with central nucleation was very similar to the histological changes observed in *mtm1* knockdown fish (Figure 3A, arrow). The proportion of fibers with central nuclei was almost similar in *mtmr12* morphant (54.5±7.1%) and *mtm1* morphant (57.8±7.1%) fish and significantly higher than normal controls (2.2±1.1%). *Mtm1-mtmr12* double knockdown fish exhibited severe muscle abnormalities with larger number of myofibers displaying sarcomeric disorganization and central nucleation (69.2±6.4%) than single morphants (Figure 3A). To evaluate if the sarcomeric defects observed in *mtmr12* morphant fish are developmental or due to degenerative changes in muscle, skeletal muscle histology was evaluated at different time points during zebrafish development. A comparison of Hematoxylin and Eosin stained skeletal muscle sections at 2 dpf and 3 dpf showed an increase in sarcomeric disorganization at 3 dpf in *mtmr12* morphant fish (Figure 3C). This suggests the sarcomeric defects seen in mtmr12 morphant fish are due to degenerative processes in disease state. The number of central nuclei per myofiber showed no significant change during development in *mtmr12* morphants (Figure 3D). A comprehensive histological analysis of Hematoxylin and Eosin stained sections of wild-type and *mtmr12* morphant showed no other histological abnormalities in other organs.

**Figure 4:**
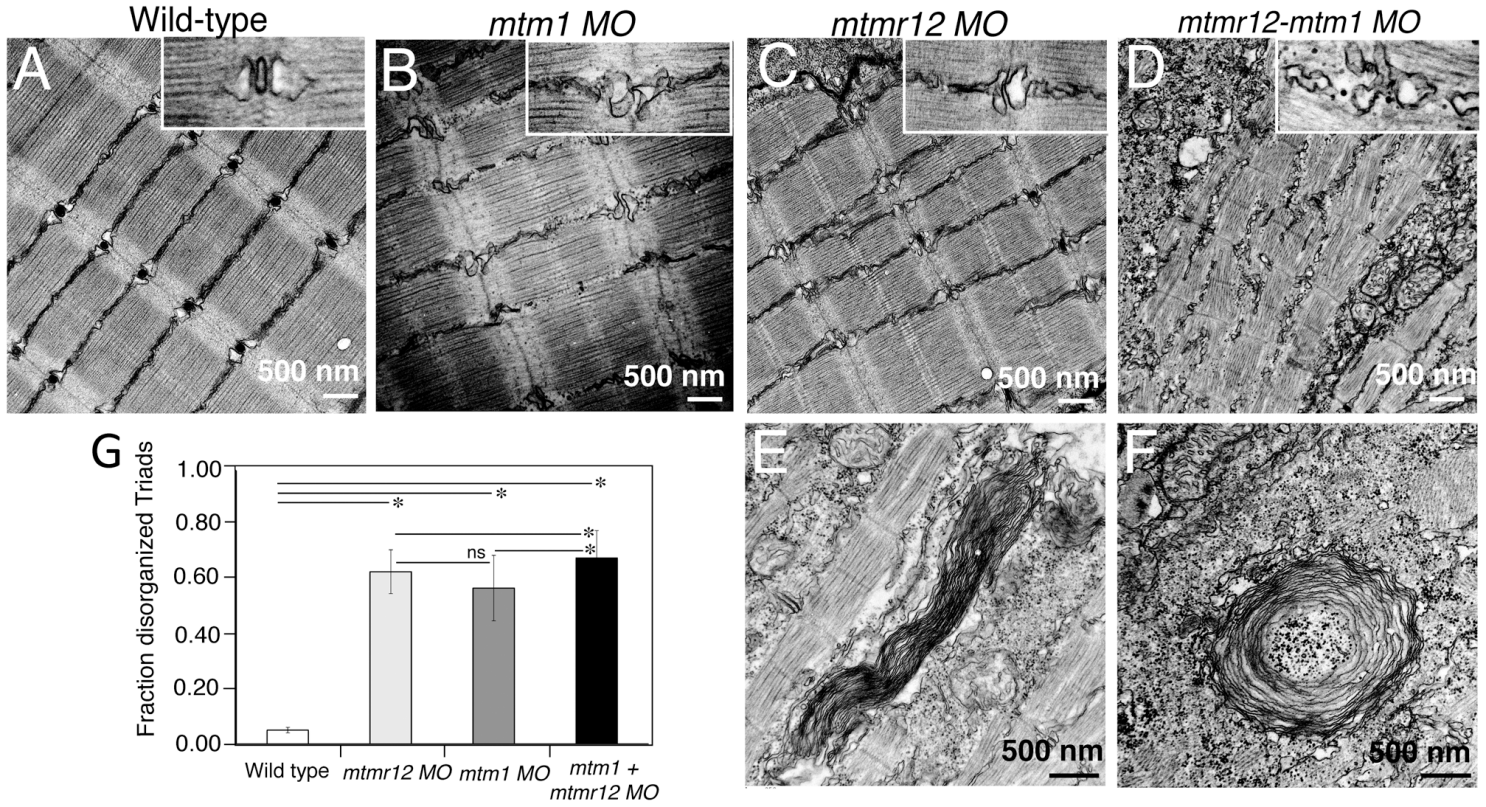
Ultrastructural abnormalities in *MTMR12* deficiency. Transmission electron micrographs of skeletal muscles in control and morphant fish. In comparison to wild-type controls (A), *mtmr12* morphant fish displayed abnormal triads (C, high magnification, inset). *mtm1* morphant muscle also had absent or disorganized triads (B). *mtmr12-mtm1* double knockdown morphants displayed exacerbated defects in muscle with many absent Z-lines in addition to disorganized triads (D inset shows high magnification view). Higher magnification examination showed whorled membranous structures in *mtmr12* and *mtmr12-mtm1* morphants (E–F). (G) Histograms represent quantification of disorganized triads of the single or double morphants. Total number of triads were counted in at least 15 myofibers within each embryo (n = 5 embryos). *P≤0.05, ns: statistically not significant.

To identify ultrastructural defects in sub-cellular compartments of skeletal muscle, transmission electron microscopy was performed at 3 dpf (Figure 4). Longitudinal views of the skeletal muscle in *mtmr12* morphant fish revealed significant myofibrillar disarray in comparison to highly organized myofibrillar structures with peripheral elongated nuclei in control fish (Figure 4A, C). Notably, skeletal muscle of *mtmr12* morphants showed an increase in absent or disorganized Triads in the myofibers in comparison to the control fish (Figure 4A, C). In addition, similar to the whorled membrane structures reported in myotubularin deficiency [13], *mtmr12* fish also exhibited whorled membrane structures in skeletal muscle (Figure 4E). *Mtm1* morphant fish displayed sarcomeric disorganization, with central nucleation and triad disorganization as previously reported for myotubularin deficiency in zebrafish, mouse and humans (Figure 4B). The ultrastructural defects in double knockdown fish were more severe than either *mtm1* or *mtmr12* knockdown fish (Figure 4D). Myofibers lacked the sarcomeric organization with absence of Z-lines in many myofibers. The numbers of disorganized triads also showed a small but significant increase in double knockdown fish (68.8±10.08%) compared to *mtm1* (63.5±8.00%) or *mtmr12* (57±12.12%) fish (p<0.005, n = 5 embryos, 15 myofibers in each embryo) (Figure 4G). Like in myotubularin and MTMR12 deficient muscles, whorled membrane structures were also observed in *mtmr12-mtm1* double knockdown zebrafish (4F). These abnormal membrane structures are seen in several types of myopathies, however, their functional role in disease pathology is not known. In a previous study on double *mtm1*-*mtmr14* knockdown, the severe phenotype was a result of an increase in autophagy in the absence of both proteins [34]. Ultrastructural examination of *mtm1*-*mtmr12* double knockdown exhibited no increase in autophagic vacuoles suggesting different pathological mechanism in two different disease states.

### Loss of MTMR12 results in reduced stability of myotubularin protein in zebrafish

**Figure 5:**
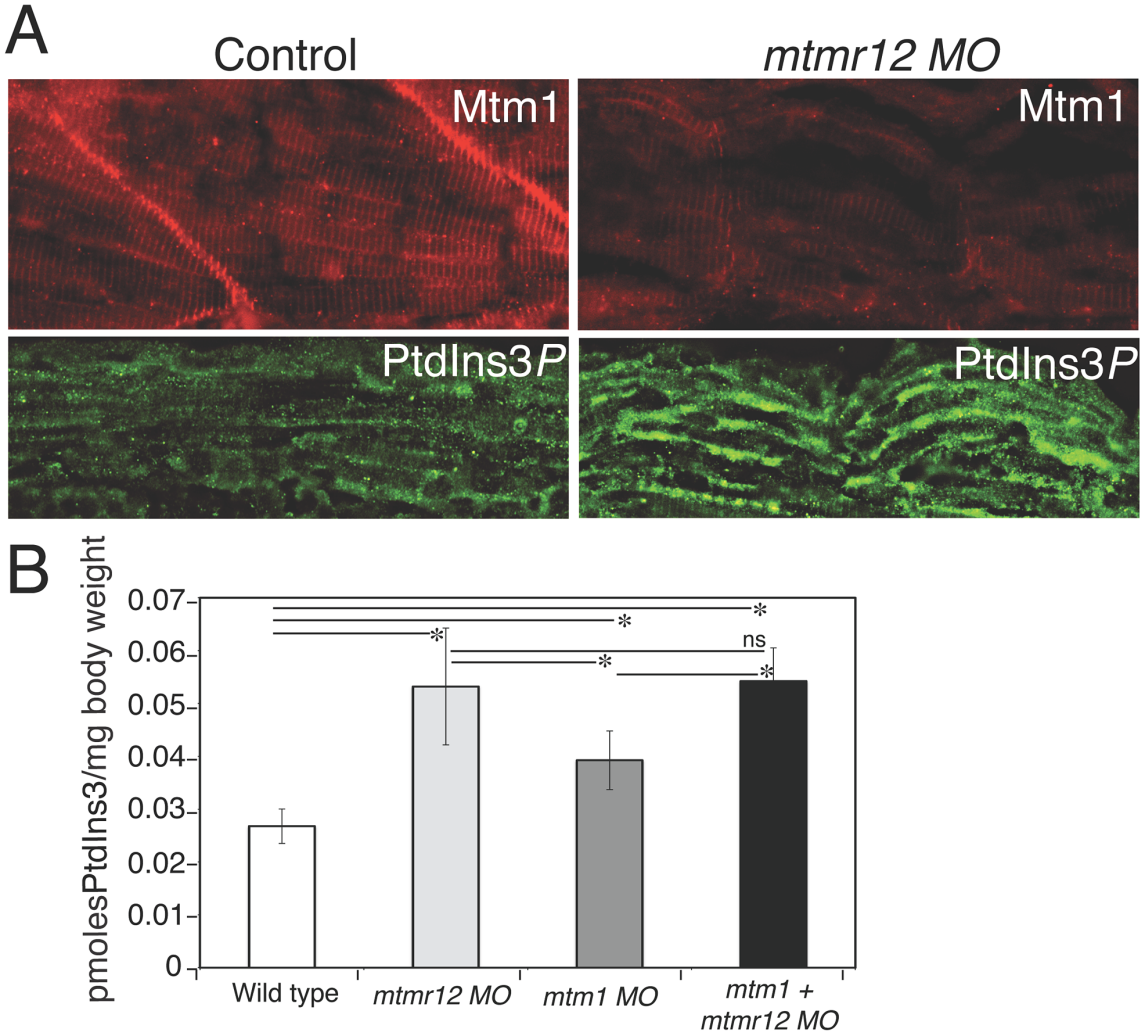
Myotubularin and PtdIns3*P* alterations in *mtmr12* morphants. (A) Immunofluorescence of control and *mtmr12* knockdown fish showed significantly decreased myotubularin staining in *mtmr12* knockdown fish in images taken under identical conditions. Immunofluorescence detection of PtdIns3*P* showed apparent increases of this myotubularin substrate in *mtmr12* morphant embryos as compared to controls. (B) PtdIns3*P* levels are increased in *mtmr12*, *mtm1* and *mtm1-mtmr12* morphant zebrafish, *P≤0.05. Total lipids were extracted from zebrafish at 3 dpf and PtdIns3*P* levels were measured using a lipid-protein overlay enzyme-linked immunosorbent assay.

Catalytically inactive myotubularin family members have been shown to regulate the activity and/or sub-cellular localization of their catalytically active interacting partners. Previous co-transfection experiments in cell culture have suggested that MTMR12 is required to regulate the subcellular localization of myotubularin [30]. Therefore, to investigate if MTMR12 controls the subcellular localization of myotubularin in zebrafish skeletal muscle, immunofluorescence studies were performed. Immunostaining of skeletal muscle of control fish with antibody against myotubularin detected a striated expression pattern of myotubularin protein corresponding to the triad compartment (Figure 5). Strikingly, highly reduced levels of myotubularin labeling were seen in morphant muscle as compared to control skeletal muscle. However, the residual myotubularin protein showed similar localization as the control fish. These data suggest that loss of MTMR12 does not affect subcellular localization of myotubularin in skeletal muscle *in vivo*.

**Figure 6:**
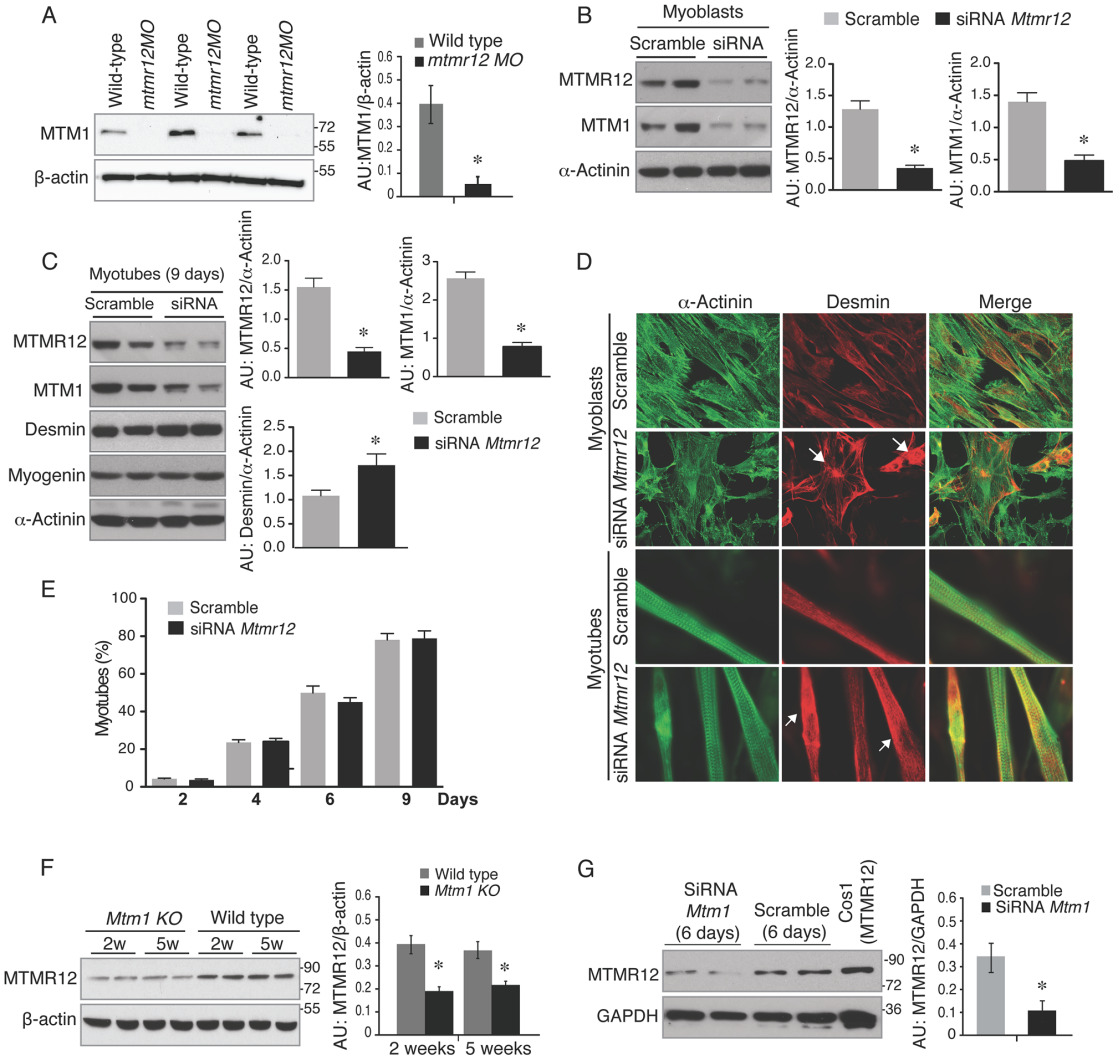
Loss of protein stability in the absence of myotubularin-MTMR12 interactions. (A) Knockdown of *mtmr12* resulted in a strong decrease of myotubularin protein in *mtmr12* morphant zebrafish at 3 dpf by western blotting. Western blot analysis was done on three independently injected clutches (n in each clutch = 50–75). The histogram at right shows normalized amounts of myotubularin in control and *mtmr12* morphant zerbrafish, *P≤0.01. (B) siRNA-mediated knockdown of *Mtmr12* in C2C12 myoblasts leads to decreased protein levels of MTM1. Histograms showed western blot quantification of MTM1 and MTMR12 with reference to α-actinin as a loading control. Data represent mean of 3 independent experiments, * P<0.05. (C) *Mtmr12* siRNA treated myoblasts were differentiated into myotubes and tested for protein expression of MTM1, MTMR12, desmin and myogenin. *Mtmr12* knockdown in myotubes leads to decreased protein levels of MTM1 and increased amounts of the intermediate filament protein desmin, but do not affect myogenin levels. α-Actinin was used as the loading control (histograms). Data represent mean of 3 independent experiments, * P<0.05. (D) Labeling of α-actinin and desmin in C2C12 myoblast and myotubes treated with *Mtmr12* siRNA or scramble control siRNA. *Mtmr12* knockdown cells showed abnormal accumulation of desmin in both stages (arrow). (E) Quantification of myotubes at 2, 4, 6 and 9 days of differentiation showed no significant differences between siRNA-*Mtmr12* cells and scramble siRNA. Data were obtained from 2 independent experiments (* P<0.05) and minimum of 100 cells per condition were counted. (F) *Mtm1* knockout mice exhibited highly reduced levels of MTMR12 protein in skeletal muscle at pre-symptomatic (2 weeks) as well as symptomatic stages (5 weeks). The histogram on right shows normalized amounts of MTMR12 in control and *Mtm1KO Mice*, *P≤0.05. (G) siRNA-based *Mtm1* knockdown in C2C12 cells led to no reduction in MTMR12 protein as seen by western blot analysis. β-actin/GAPDH were used as loading controls in western blots. The histogram at right shows normalized amounts of MTMR12 in control and *Mtm1* knockdown cell line, *P≤0.05.

These observations suggest that MTMR12 is likely involved in regulating the stability of myotubularin. To address this point, we quantified myotubularin protein levels in *mtmr12* morphant fish. Western blot analysis, performed in three independent groups of embryos injected with *mtmr12* morpholino, revealed a ∼90% reduction in myotubularin levels in *mtmr12* morphant fish in comparison to the controls (Figure 6A). This suggests that myotubularin-MTMR12 interactions result in stabilization of myotubularin in zebrafish skeletal muscle. To investigate whether MTMR12 regulates myotubularin levels in a mammalian system, *Mtmr12* siRNA C2C12 cell lines were created. SiRNA-mediated knockdown of *Mtmr12* in C2C12 myoblasts or differentiated myotubes resulted in decreased levels of myotubularin protein compared to scrambled control siRNAs (Figure 6B and 6C). MTMR12 deficient C2C12 myotubes also mimicked cellular changes previously seen in *Mtm1* knockdown C2C12 cells [35]. *Mtmr12* knockdown in C2C12 cells resulted in an increase in levels of the intermediate filament protein desmin which were associated with abnormal filament shape (arrow) in both myoblasts and myotubes (Figure 6C and 6D). However, no change in differentiation markers such as myogenin or myotube formation was observed in *Mtmr12* knockdown cells (Figure 6C and 6E) suggesting that Mtmr12 deficiency does not affect the differentiation program in C2C12 cells.

To determine whether MTMR12 protein stability is regulated by myotubularin, MTMR12 levels were assessed in *Mtm1* knockout mice and *Mtm1* knockdown C2C12 cells (Figure 6F–G). In *Mtm1* knockout mice, expression of MTMR12 was reduced in skeletal muscle in pre-symptomatic (2 weeks) as well as in symptomatic phases of disease progression (5 weeks) (Figure 6F). Similarly, in *Mtm1* knockdown cell lines, a significant decrease in MTMR12 expression levels was also observed (Figure 6G). Combined, these data support the notion that the MTMR12-myotubularin interaction enhances stability of the complex in muscle cells *in vitro* and in skeletal muscle *in vivo*.

The only known and well-characterized biochemical functions of myotubularin related proteins is the dephosphrylation of phospholipids. Previous data have shown that *Mtm1* mice and *mtm1* zebrafish morphants display an increase in PtdIns3*P* levels (the substrate of myotubularin) [11], [13] To investigate if *mtmr12* knockdown also affects PItdIns3*P* levels in skeletal muscle as seen in myotubular myopathy, PtdIns3*P* staining was performed on control and *mtmr12* morphant skeletal muscle. Indirect immunofluorescence on MTMR12-deficient muscle showed an increase in PtdIns3*P* staining compared to control suggesting that the reduction of myotubularin levels is paralleled by an overall decrease in its enzymatic activity (Figure 5). To quantify the levels of PtdIns3*P* in the absence of either myotubularin or MTMR12 or both, a lipid-protein overlay PtdIns3*P* ELISA was performed on lipid extracts from 3 dpf zebrafish morphant fish. As shown previously [11], [13], absence of myotubularin resulted in an increase in PtdIns3*P* levels in *mtm1* morphant embryos (Figure 5B). In the absence of MTMR12 or both myotubularin and MTMR12, a significant increase in PtdIns3*P* levels were seen over levels in both control and myotubularin morphant fish. This suggests that in addition to regulating myotubularin activity, MTMR12 may also be regulating the functions of other PtdIns3*P* phosphatases.

### Over-expression of *MTM1* improves skeletal muscle function in *Mtmr12* morphants

**Figure 7:**
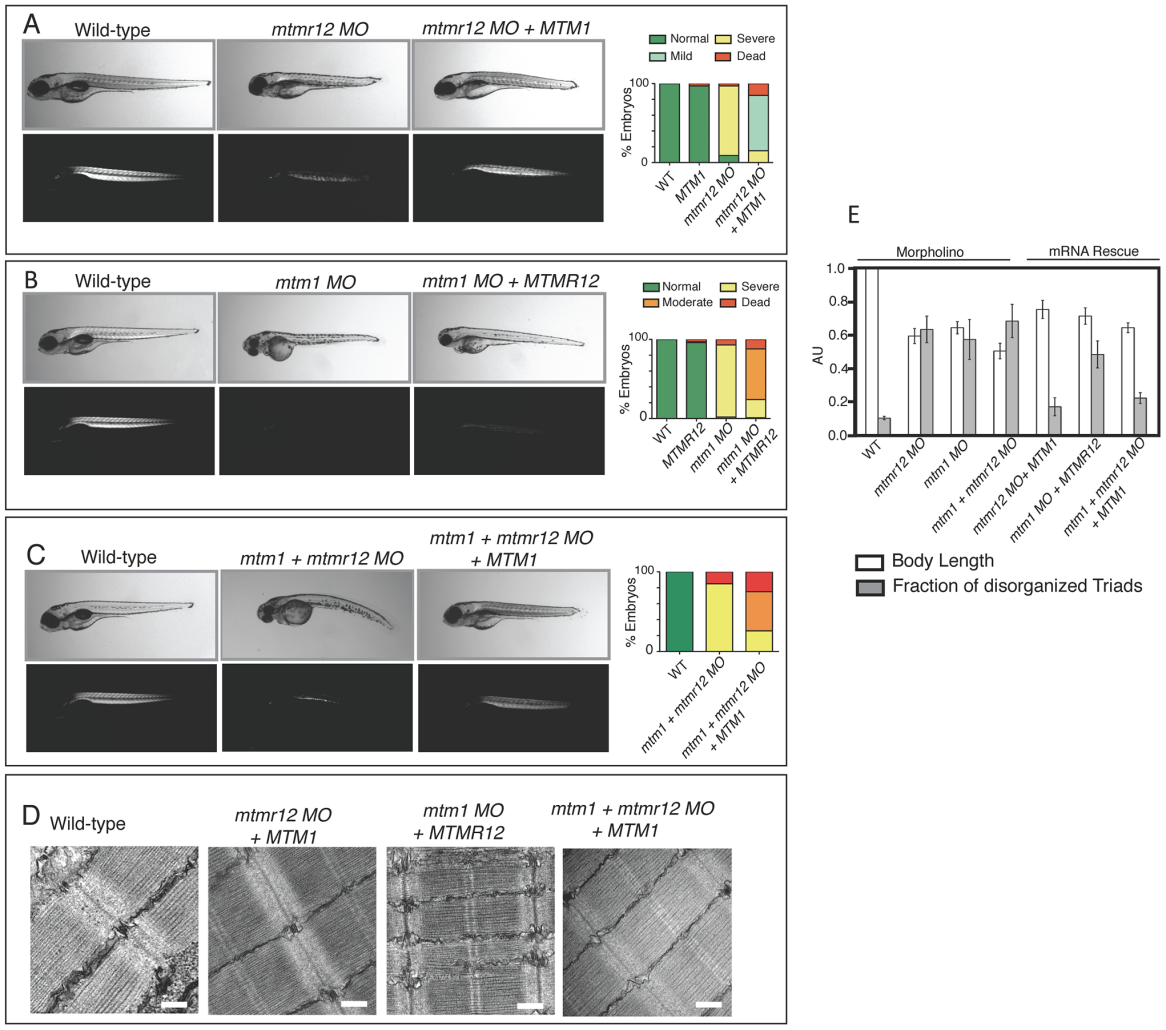
Rescue of *mtmr12* morphant phenotypes by *MTM1*. The ability of human *MTM1* or *MTMR12* transcripts to rescue abnormalities seen in morphant zebrafish was classified in to phenotypic index of five groups: Normal, mild, moderate, severe and dead, described in the table depending on body length, birefringence and ultrastructure of skeletal muscle. (A) Polarized light microscopy of 3 dpf live embryos showed that birefringence of *mtmr12* morphant embryos increased significantly upon overexpression of human *MTM1* mRNA. (B) Overexpression of human *MTMR12* mRNA in *mtm1* morphant fish resulted in a mild rescue of skeletal muscle defects as seen by birefringence of zebrafish embryos. (C) Overexpression of human *MTM1* mRNA in *mtm1-mtmr12* morphant fish resulted in a moderate rescue of skeletal muscle defects as seen by birefringence of zebrafish embryos. (D) Electron microscopy showed normal skeletal muscle structure of *mtmr12* and *mtm1-mtmr12* morphant fish rescued with *MTM1* mRNA but displayed disorganized triads in *mtm1* morphants that were rescued with *MTMR12* mRNA. (E) Quantification of the body length and disorganized triads in morphant and rescued fish. Body length was measured in 10–15 embryos in each group. Total number of triads were counted in at least 15 myofibers within each embryo (n = 5 embryos). P≤0.05.

*Mtmr12* morphant fish displayed reduced levels of myotubularin protein and similar pathological changes to those seen in myotubularin deficiency, suggesting that the ability of MTMR12 to stabilize endogenous myotubularin levels *in vivo* may be a primary physiological role. These findings gave rise to the intriguing possibility that overexpression of myotubularin may be able to reverse the phenotypes associated with MTMR12 deficiency in zebrafish. To test this hypothesis, we overexpressed exogenous human *MTM1* mRNA to investigate its ability to rescue the muscle phenotype in *mtmr12* morphant fish and evaluated the resulting skeletal muscle phenotypes by birefringence assay and electron microscopy (Figure 7). Overexpression of *MTM1* mRNA in *mtmr12* morphant fish restored the birefringence to almost normal levels (Figure 7A). While the skeletal muscle birefringence was drastically improved, the length of zebrafish embryos was smaller (75±5.4% of control fish) suggesting MTMR12 may have additional functions independent of myotubularin. The ultra-structure of skeletal muscle was also significantly improved with a decrease in fraction of abnormal triads. This result strengthens the finding that an important role of MTMR12 is to provide stability to myotubularin protein and hence regulate its function. The *mtm1-mtmr12* double knockdown fish exhibited severe muscle defects consistent with the idea that MTMR12 may have independent functional roles in addition to providing stability to myotubularin. This was investigated by overexpressing *MTM1* RNA in *mtm1-mtmr12* double knockdown fish. Overexpression of *MTM1* resulted in a moderate improvement in birefringence as well as skeletal muscle pathology in double knockdowns as quantified by number of normal triads in skeletal muscle of rescued fish. Nevertheless, rescued fish were still smaller in size (0.64±0.029) than wild-type controls (1.00±0), consistent with the notion that MTMR12 may have functions independent of MTM1 *in vivo* (Figure 7 C and E).

To test the ability of MTMR12 to rescue the phenotypes associated with myotubularin deficiency in skeletal muscle, human *MTMR12* RNA was overexpressed in *mtm1* morphant fish. This resulted in partial rescue of the muscle phenotype in these fish as seen by an increase in birefringence and body length (71±4.9% of normal controls) of *mtm1* morphant fish rescued with *MTMR12* mRNA versus *mtm1* morphant (64±3.68% of normal controls), however, without any significant reduction in disorganized triads (Figure 7B, C and E). These observations suggest that it is the missing catalytic activity or other gene-specific function of myotubularin that is primarily responsible for the pathology of XLMTM.

### Myotubularin-MTMR12 interactions are perturbed in XLMTM

**Figure 8:**
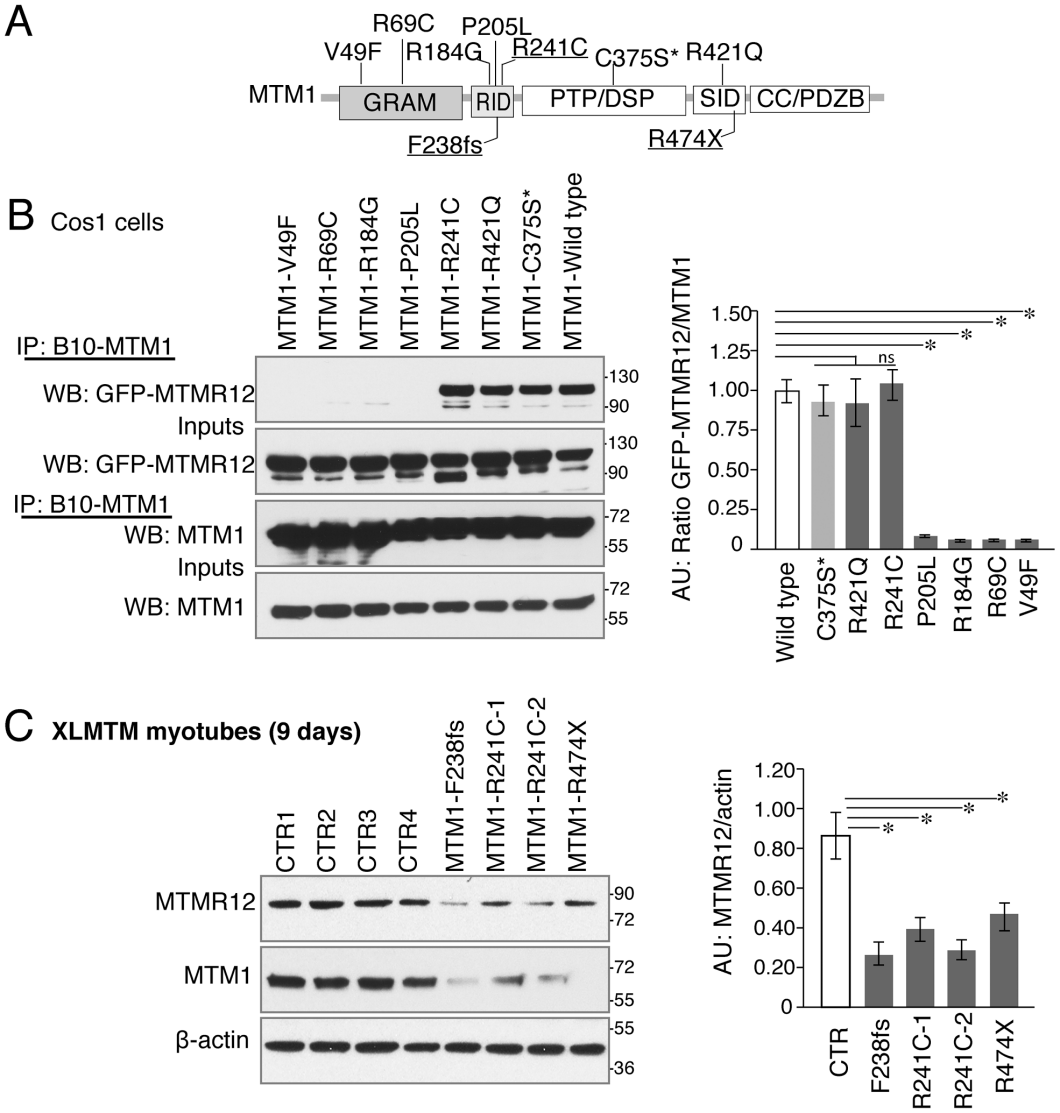
Myotubularin-MTMR12 interactions in XLMTM. (A) Schematic diagram of different domains of myotubularin protein displaying representative pathogenic mutations found in XLMTM patients or an artificial inactivating mutation C375S* (GRAM, N terminal lipid or protein interacting domain; RID, putative membrane targeting motif; PTP/DSP, phosphatase domain; SID, protein-protein interacting domain; CC, coiled-coil domain; PDZB, PDZ binding site). (B) Wild-type or mutant MTM1-B10 fusion proteins with indicated missense mutations and wild-type MTMR12-GFP proteins were overexpressed in Cos1 cells. Immunoprecipitation of protein extracts with anti-B10 tag antibody showed that mutations on GRAM or RID domains disrupt the interactions between MTM1 and MTMR12. (C) Western blotting of XLMTM patient myotubes showed that mutants that decrease the stability of myotubularin protein also results in a reduction of MTMR12 levels. Histograms depict the western quantification for panels (B) and (C). Asterisks indicate statistically significant differences from measurements of wild type controls, P≤0.05.

The significance of MTM1-MTMR12 interactions was investigated in the human neuromuscular disease XLMTM. In XLMTM, more than 200 mutations have been reported that are distributed on different domains of the myotubularin protein (http://www.dmd.nl/). In addition to nonsense mutations, a large number of missense mutations in various myotubularin domains have been shown to be pathogenic. To test, if disease causing mutations in myotubularin affect its interaction with MTMR12, interactions between mutant myotubularins and wild-type MTMR12 were examined. A series of human myotubularin proteins modeling human missense mutations in different domains were constructed (Figure 8A). Exogenous wild type or mutant myotubularins were co-expressed with MTMR12-GFP protein in Cos7 cells. Immunoprecipitation of protein extracts with a myotubularin specific antibody revealed that missense mutations in the GRAM or RID domains abolished the interaction of myotubularin with MTMR12 (Figure 8B). As over-expression in cell culture may not represent the physiological milieu, myotubes from XLMTM patients were also used to test if the pathogenic mutations on myotubularin protein affect myotubularin-MTMR12 interactions. Examination of XLMTM patient myotubes showed all mutations tested resulted in highly reduced levels of myotubularin compared to control samples. In addition, MTMR12 levels were also reduced in these patients suggesting an overall perturbation of myotubularin-MTMR12 complexes in XLMTM (Figure 8C).

## Discussion

Studies presented in this work were aimed at gaining insights into the molecular regulatory mechanism(s) of myotubularin function *in vivo*. Previous studies have shown that MTMR12 is an interacting partner of myotubularin *in vitro* [30], [33]. Here, we show that absence of catalytically inactive phosphatase MTMR12 protein resulted in skeletal muscle myopathy and pathological changes similar to XLMTM due to abrogation of protein-protein interactions between myotubularin and MTMR12 resulting in reduced stability and loss of myotubularin protein function.

The interaction between myotubularin and MTMR12 in skeletal muscle and co-localization at the triad certainly suggests that they might be functioning together in similar biological processes in muscle cells. This hypothesis is supported by knockdown studies on the *mtmr12* gene in zebrafish that resulted in myopathic muscle in the affected fish similar to the myotubularin deficient zebrafish model [13]. To understand if these proteins function in similar biological processes or play roles in other processes independent of each other, *mtm1*-*mtmr12* double knockdown zebrafish were created. The phenotype of double knockdown zebrafish was more severe than fish deficient in either myotubularin or MTMR12 alone. Further, inability of MTM1 to rescue all the defects observed in double knock-down fish suggests that these proteins may play additional functions that are independent of each other either by interacting with other proteins within or outside of the myotubularin family. Previous studies have shown that in addition to myotubularin, MTMR12 also interacts with another catalytically active member, MTMR2, by co-immunoprecipitation and yeast two hybrid interactions [29], [30]. Therefore, future studies on identification of protein complexes of myotubularin and MTMR12 proteins may help in identifying other pathways that are regulated by these proteins.

The presence of similar pathological changes in *mtmr12* knockdown and XLMTM muscles, such as myofibrillar disarray, excessive central nucleation, triad disorganization and presence of whorled membranous structures, suggests that *mtmr12* is a crucial regulator of disease pathology in XLMTM. Moreover, these pathological changes in zebrafish manifest early in zebrafish development (2–3 dpf), similar to pathological changes seen in human patients. As loss of *mtmr12* resulted in clinical symptoms similar to those associated with centronuclear myopathies, *MTMR12* represents an excellent candidate gene for patients with centronuclear myopathy but unknown genetic diagnosis. However, sequencing of 108 such cases failed to identify any pathogenic mutations in *MTMR12*, suggesting *MTMR12* may account for a small subset of patients or is mutated in a clinically different disease, perhaps with a skeletal muscle component, but related also to other functions of MTMR12 (V.A. Gupta, unpublished data). In our cellular models of MTMR12 deficiency, highly reduced levels of MTM1 were observed. Therefore, any genetically unknown cases exhibiting low levels of myotubularin without any *MTM1* mutations may also be good candidates for testing for *MTMR12* mutations. Next generation sequencing technologies have been exhibited great promise in identifying rare gene variants and may yet identify *MTMR12* mutations in the future. Regardless, these studies show a crucial role for MTMR12 function in XLMTM disease pathology.

Complex formation between some catalytically inactive and active partners in the myotubularin family has been shown to increase the activity of the catalytically active binding partner either by recruitment to specific membrane subdomains rich in lipid substrate or by increasing the allosteric activity. In the absence of MTMR12 an increase in PtdIns3*P* was observed suggesting a decrease in myotubularin and/or another partner's enzymatic activity. However, unlike other catalytically active-inactive pairs, MTMR12 primarily regulates the function of myotubularin protein by affecting protein levels instead of modulating the enzymatic activity [22], [36]. Interestingly, PtdIns3*P* levels were higher in *mtmr12* morphant fish in comparison to *mtm1* morphant fish, suggesting that MTMR12 may function in regulating the enzymatic activity/protein stability of other phospholipid phosphatases in cells. In the absence of MTMR12, highly reduced levels of myotubularin protein was observed suggesting that protein-protein interactions between these proteins are required for maintaining myotubularin stability. Similarly, decreased levels of MTMR12 were seen in myotubularin deficient cells and mice suggesting that stability of MTMR12 is also dependent on the interaction with myotubularin.

The abnormalities observed in MTMR12 deficient fish appear mainly due to loss of function of myotubularin as overexpression of *MTM1* mRNA dramatically improved the skeletal muscle defects observed in *mtmr12* morphant embryos while overexpression of catalytically inactive *MTMR12* mildly improved muscle pathology in *mtm1* morphant embryos. This suggests that enzymatic activity of myotubularin is required for its protein function, which was also seen, in a previous study where myotubularin deficiency could be rescued by catalytically active MTMR1 and MTMR2 [13]. Recent work also suggests that several but not all structural abnormalities observed in myotubularin deficiency can be rescued by over-expression of a catalytically inactive form of myotubularin [18]. As the catalytically inactive MTMR12 only partially but significantly rescues myotubularin function, it supports these recent findings [18] and suggests that MTMR proteins partially compensate for the lack of MTM1 through functions independent of their catalytic activity.

This implies that the inability of catalytically inactive MTMR12 to rescue myotubularin function is not only due to the lack of phosphatase activity but could also be due to other properties of myotubularin not compensated by all MTMRs. MTMR12 also interacts with another catalytically active member, MTMR2, by co-immunoprecipitation and yeast two hybrid [29], [30]. Further MTMR2 and MTMR13 also interact by direct protein-protein binding. Mutations in genes encoding MTMR2 and MTMR13 have both been associated with Charcot-Marie-Tooth (CMT) disease raising the possibility that MTMR12 deficiency may result in neurological defects such as those seen in CMT. *Mtmr2* as well as *Mtmr13* knockout mice exhibit a progressive neuropathy that becomes evident much later in the life span of these mice (∼6 months) [27], [37]. In comparison, no such neurological defects were seen in *mtmr12* morphant fish, which could be due to early mortality of these embryos (between 3–5 dpf), before such changes become evident. Finally, it is worth noting that the ability of human *MTM1* mRNA to rescue zebrafish *mtm1* morphants reinforces the notion that these orthologues have been functionally conserved despite their considerable evolutionary distance, validating the relevance of the zebrafish model to studies of human XLMTM.

Studying the interaction of MTMR12 with myotubularins modeling various human mutations illuminates the role of myotubularin-MTMR12 interactions in XLMTM. Missense mutations in the N-terminal GRAM and RID domains resulted in abolishment of interaction between myotubularin and MTMR12. This was a surprising finding as previous studies have shown the myotubularin family members interact with each other through their SID or coiled-coil domains. It was also shown that an isoform of MTMR12 lacking the SID domain showed no interaction with myotubularin protein [30]. One reason for this discrepancy is that many of the previous studies have been performed using deletion constructs of different domains that may have a different effect on protein confirmation and thus its interactions than the missense changes we studied. Nonetheless a study of interactions between MTMR6 and KCa3.1 proteins has shown that along with the coiled-coil domain, the PH-GRAM domain is also required for protein-protein interactions [38]. Similarly, oligomerization of MTMR2-MTMR13 in a complex occurs independent of coiled-coil domains [39]. Analysis of XLMTM patient myotubes showed that many mutations of myotubularin that result in low levels of myotubularin protein also lead to decreases in MTMR12 levels. Many patients with these types of myotubularin mutations are described with severe phenotypes and a further reduction of MTMR12 may exacerbate the clinical severity as seen in myotubularin-MTMR12 double knockdown zebrafish.

**Figure 9:**
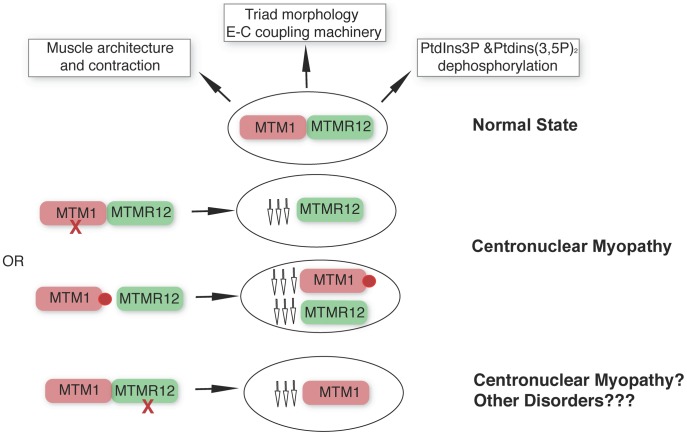
MTM1-MTMR12 interactions in normal and disease states. Under normal conditions, MTM1 and MTMR12 interact in skeletal muscle and regulate skeletal muscle architecture and function. Loss of function mutations of *MTM1* (red cross) in skeletal muscle are associated with centronuclear myopathy and with a secondary reduction in MTMR12 levels. In centronuclear myopathy, disease causing missense mutations (red circle) that disrupt interactions between MTM1 and MTMR12 result in decreased stability of myotubularin causing myotubular myopathy associated with reduced levels of MTMR12. Loss of *MTMR12* in zebrafish and mammalian cells, results in decreased levels of myotubularin resulting in pathological changes similar to centronuclear myopathy.

Apart from the type III intermediate filament desmin [35], our study identifies a second interactor of myotubularin in skeletal muscle and underlines the concept that protein-interactions between myotubularin and MTMR12 are crucial for disease pathology in XLMTM. We propose a model whereby disruption of interactions between MTM1-MTMM12 results in destabilization of both partners in the complex, leading to centronuclear myopathy (Figure 9). As loss of MTMR12 results in reduction of myotubularin, we suspect that primary mutations of MTMR12 may result in centronuclear or related myopathies. As protein interactions play critical roles in almost all biological processes, efforts are currently focused on identifying drugs that can stabilize protein-protein interactions [40]. Stabilizing protein-protein interaction between myotubularin and MTMR12 may result in restoration of normal skeletal muscle function in XLMTM patients with certain missense mutations of myotubularin.

## Materials and Methods

### Fish and embryo maintenance

Fish were bred and maintained as described previously [41]. Control embryos were obtained from the Oregon AB line and were staged by hours (hpf) or days (dpf) post fertilization at 28.5°C. All animal work was performed with approval from the Boston Children's Hospital Animal Care and Use Committee.

### Ethics statement for mouse work

Animals were housed in a temperature-controlled room (19–22°C) with a 12:12 hr light/dark cycle. Mice were humanely killed by CO_2_ inhalation followed by cervical dislocation, according to national and European legislations on animal experimentation.

### Whole mount in situ hybridization

Isoform-specific riboprobes were constructed from the 3′UTRs of *mtm1* and *mtmr12* using adult zebrafish RNA. Total RNA was extracted from adult zebrafish muscle tissue using Trizol (Invitrogen, Carlsbad, CA, USA). cDNAs were synthesized using superscript RT-PCR system (Invitrogen) and cloned in pZEM7Z(+) using XhoI and BamHI sites respectively. Sense or antisense digoxigenin-labeled riboprobes were synthesized by *in vitro* transcription using dig-labeling kits (Roche Applied Sciences, Indianapolis, IN, USA). Whole mount *in situ* hybridization was performed as described [42]. Imaging was performed using a Nikon SMZ1500 microscope with a Spot camera system.

### Morpholino knockdown and mRNA rescue

Two splice site blocking morpholinos targeting different exon-intron boundaries, and a translational blocking morpholino, were designed to knockdown zebrafish *mtm1* or *mtmr12* transcripts (Genetools, Philomath, OR, USA). The morpholino sequences are *mtm1* (translational): AGCCAGACCCTCGTCGAAAAGTCAT, mtmr12 (translational): CTCCTCCGCTCCCCAAACTCAACAT, mtmr12 (exon3-intron3): GCCCGGTCAACTGTCCTTACCATCT. Morpholino against human β-globin was used as a negative control for all injections. Morpholinos were dissolved in 1X Danieau buffer and 1–2 nl (1–10 ng) were injected into 1cell embryos.

For rescue experiments, full-length human *MTM1* and *MTMR12* cDNAs were cloned in to a PCSDest destination vector (a gift from Nathan Lawson) using Gateway technology (Invitrogen, Carlsbad, CA, USA). mRNA was synthesized *in vitro* using mMessage kits (Ambion, Austin, TX, USA). 50–200 pg of mRNA was injected into embryos at the 1 cell stage.

### Immunofluorescence

Indirect immunofluorescence staining was performed on zebrafish frozen sections as described previously [43]. Primary antibodies used for zebrafish experiments were rabbit anti myotubularin HPA010008 (Sigma-Aldrich, St. Louis, MO, USA) and anti PtdIns3*P*, Z-P345b (Echelon Bioscience, Salt Lake City, UT, USA). For mouse muscle staining, tibialis anterior sections (8 µM) were labeled successively with the anti-MTM1 polyclonal antibody (2827) [35] and the anti-MTMR12 antibody (GTX119163, GeneTex Inc., Irvine, CA, USA). Briefly, after permeabilization and blocking, the anti-MTM1 antibody was applied (diluted at 1/500) on section for 2 h at RT. After washing steps, sections were incubated with the secondary antibody coupled to Alexa fluor 488 (Invitrogen) for 45 min followed by a second step of washing cycles and a blocking step. Then, the anti-MTMR12 antibody was applied on section for 2 h at RT and revealed by the incubation with a secondary antibody coupled to Alexa fluor 594 (Invitrogen). After final washing, sections were fixed, mounted and observed under confocal microscope (Leica SP2 MP confocal microscope). The monoclonal antibody against RyR1 (1C3) was a gift from Dr Isabelle Marty (Grenoble, France). The mouse anti α-actinin antibody (clone EA-53) was from Sigma (Sigma-Aldrich, St. Louis, MO, USA).

### Western blotting

Zebrafish embryos were homogenized in a buffer containing Tris-Cl (20 mM, pH 7.6), NaCl (50 mM), EDTA (1 mM), NP-40 (0.1%) and complete protease inhibitor cocktail (Roche Applied Sciences). Western blotting was performed as described previously [43]. Primary antibodies used were mouse monoclonal anti-myotubularin 1G1 [19], rabbit polyclonal anti-myotubularin R2827 [44] and mouse anti-β-actin clone AC-15 (Sigma-Aldrich, A5441 at 1∶2000). Mouse monoclonal antibodies for Desmin and the sarcomeric α-actinin were purchased from Sigma-Aldrich (clone D33 and Clone EA-53, respectively). The mouse monoclonal antibody for myogenin was from R&D system (Clone 671037). Protein bands were quantified using Quantity One software (Biorad, Hercules, CA, USA).

### Cell culture and siRNA

C2C12 cells were cultured in proliferation medium (Dulbecco medium supplemented with 20% FCS and 400 U/ml of Gentamycin) and differentiation was enhanced by decreasing the FCS in the media to 2% for 1 day and then accelerated by replacing the FCS by 5% Horse serum. Cells were kept in differentiation medium for 9 days with medium replacement every 2 days. For siRNA experiments the Accel Smart Pool siRNA (Thermo Scientific, Dharmacon) were used to knockdown Mtmr12 in C2C12. Myoblasts (at 30–40% of confluence) were washed with PBS and incubated with siRNA medium containing permeable Accel siRNA pool against Mtmr12 (CCAGCAGUAUAGAGGAAUA, GCGCUAUUUACGUUGGAUU, CCCGUGGGUUUAUAUAUUG, GGAUUAAGCUAUUAGACUG) or scrambled control siRNAs diluted to the appropriate concentration. After 72 hours, cells were washed with PBS and incubated with proliferation medium or differentiation medium and left for 9 days in order to obtain myotubes. Myotubes (containing min 2 nuclei) were counted blindly under the microscope (bright field) at different stages and minimums of 100 cells per well were counted.

### GST pull down and co-immunoprecipitation (co-IP) assays

*MTMR12* constructs were transferred to Gateway destination vectors for eukaryotic expression (pSG5 from Agilent Technologies (Santa Clara, CA, USA), with tag corresponding to the B10 epitope of estrogen receptor or GFP) and pSG5-MTM1-B10 and pcDNA3.1-MTM1 were used for co-immunoprecipitation assays in Cos1 cells. For GST-pull down experiment, *MTM1* cDNA was inserted into the prokaryotic expression vector (pGex4T3, Invitrogen, Carlsbad, CA, USA). Myotubularin recombinant proteins were produced in the BL21-Rosetta 2 strain (Novagen, Billerica, MA, USA); GST fusion proteins were purified and coupled to glutathione sepharose beads as described before [35]. pSG5-MTMR12-B10 was translated *in vitro* according to the manufacture protocol (TNT coupled reticulocyte lysate System, Promega, Madison, WI, USA). Resulted translated protein was diluted in Co-IP buffer: (50 mM Tris-Cl pH 7.5, 100 mM NaCl, 5 mM EDTA, 5 mM EGTA, 1 mM DTT, 0,5% Triton X-100, 2 mM PMSF) supplemented with complete protease inhibitor tablet (Roche Applied Sciences, Indianapolis, IN, USA) and 1 mM Leupeptin and 1 mM pepstatin (Sigma, St. Louis, MO, USA). Homogenates were centrifuged at 14.000×*g* and pull down was performed as previously described [35]. GST coupled beads was used as negative control. For Co-IP Cos7 cells were transiently transfected with pcDNA3.1-MTM1 and pSG5-MTMR12-B10 constructs or with pSG5-MTM1-B10 and pSG5-MTMR12-GFP for 24 hours and homogenized in ice-cold lysis buffer (10 mM Tris-Cl, pH 7.6; 140 mM NaCl; 5 mM EDTA; 5 mM EGTA; 0.5% [v/v] Triton X-100; and 2 mM PMSF). Homogenates were incubated with mouse monoclonal anti-myotubularin 1G1 [19], or B10 epitope (Mab anti-B10, IGBMC, Illkirch, France) [35]. Interacting proteins were analysed by Western blot as mentioned before.

The panel of amino acid changes was engineered by PCR-based mutagenesis from the cDNA encoding the wild-type protein (MTM1) using PFU DNA polymerase (Agilent technologies). All constructs were verified by sequencing.

Co-IP experiments in muscle were performed from fresh murine tibialis anterior muscles that were dissected and homogenized with a dounce homogenizer in ice-cold co-IP buffer (50 mM Tris-Cl, pH 7.5; 100 mM NaCl; 5 mM EDTA; 5 mM EGTA; 1 mM DTT; 0.5% Triton X-100; and 2 mM PMSF) supplemented with 0.05% (w/v) SDS. Lysates were centrifuged at 14.000×*g* at 4°C and precleared with 50 µl of G-sepharose beads (GE Healthcare) and subsequently incubated with antibodies of interest for 12–24 hours at 4°C. Protein G-sepharose beads (50 ml) were then added for 4 hours at 4°C to capture the immune complexes. Beads were washed 4 times with co-IP buffer and 1 time with high stringency co-IP buffer (with 300 mM NaCl). For all experiments, two negative controls consisted of a sample lacking the primary antibody (Beads) and a sample incubated with IgG. Resulting immune-bound complexes were eluted in Laemmli buffer and analysed by SDS-PAGE and Western blotting.

### PtdIns3P ELISA

PtdIns3*P* mass ELISA was performed on lipid extracts from whole zebrafish embryos from 3 independently injected clutches (number of embryos in each clutch = 50) following manufacturer's recommendations (Echelon Biosciences, Salt Lake City, UT, USA). Extracted lipids were resuspended in PBS-T with 3% protein stabilizer and then spotted on PtdIns3*P* Mass ELISA plate. Following ELISA, PtdIns3*P* levels were detected by measuring absorbance at 450 nm on a plate reader. Specific amounts were determined by comparison of values to a standard curve generated with simultaneous readings of known amounts of PtdIns3*P*.

### RT-PCR

To detect *mtmr12* knockdown in 3 dpf zebrafish embryos, RNA was prepared using RNeasy fibrous tissue mini kits (Qiagen, Valencia, CA, USA). cDNA was prepared using high capacity RNA-to-cDNA mastermix kits (Ambion, Austin, TX, USA). RT- PCR was performed using equal amounts of RNA from control and *mtmr12* morphant zebrafish. To measure *Mtmr12* and *Mtm1* expression levels in C2C12 cells, total RNA was purified from C2C12 (from 0 to 9 days of differentiation) cells using Trizol reagent (Invitrogen) according to manufacturer's instructions. cDNAs were synthesized from 2 to 5 µg of total RNA using Superscript II reverse transcriptase (Invitrogen) and random hexamers. Quantitative PCR amplification of cDNAs was performed on Light-Cycler 480 and Light-Cycler 24 instruments (Roche Applied Sciences) using 58°C as melting temperature. *Gapdh* gene expression was used as control as expression of this gene varies little during C2C12 cell differentiation. Primers used were: *Mtm1* (F: catgcgtcacttggaactgtgg, R : gcaattcctcgagcctcttt), *Mtmr12* (F: tgtctgaggtacacaaaggag, R: agccttcattcacactcactg) and *Gapdh* (F: agctttccagaggggccatccaca, R : ccagtatgactccactcacggcaa).

### Electron microscopy

Zebrafish embryos were fixed in formaldehyde-glutaraldehyde-picric acid in cacodylate buffer overnight at 4°C followed by osmication and uranyl acetate staining. Subsequently, embryos were dehydrated in a series of ethanol washes and finally embedded in TAAB epon (Marivac Ltd., Nova Scotia, Canada). 95 nm sections were cut with a Leica Ultracut microtome, picked up on 100 m Formvar coated Cu grids and stained with 0.2% Lead Citrate. Sections were viewed and imaged under a Philips Tecnai BioTwin Spirit Electron Microscope (Electron Microscopy Core, Harvard Medical School).

### Quantification and statistical analysis

Centrally nucleated myofibers were quantified by analyzing serial sections from 6 different embryos. The relative number of centrally nucleated fibers in the middle somites (10–13) were counted. Total number of triads were counted in at least 15 myofibers within each embryo (n = 5 embryos). Body length was measured in 10–15 embryos in each group. Data were statistically analyzed by parametric Student *t*-test (two tailed) and were considered significant when P<0.05. All data analyses were performed using XLSTAT software.

## Supporting Information

Movie S1 Touch evoke response assay in wild-type (fish1), *mtmr12* (fish2), *mtm1* (fish3) and *mtm1-mtmr12* (fish4) morphant embryos at 3 dpf.
(MP4)
